# Molecular characterization of ascaridoid parasites from captive wild carnivores in China using ribosomal and mitochondrial sequences

**DOI:** 10.1186/s13071-020-04254-4

**Published:** 2020-07-29

**Authors:** Yue Xie, Yingxin Li, Xiaobin Gu, Yunjian Liu, Xuan Zhou, Lu Wang, Ran He, Xuerong Peng, Guangyou Yang

**Affiliations:** 1 Department of Parasitology, College of Veterinary Medicine, Sichuan Agricultural University, Chengdu, 611130 China; 2 Institute of Animal Genetics and Breeding, College of Animal Science and Technology, Sichuan Agricultural University, Chengdu, 611130 China; 3 Department of Chemistry, College of Life and Basic Science, Sichuan Agricultural University, Chengdu, 611130 China

**Keywords:** *Toxocara*, *Toxascaris*, Wild canids and felids, Nuclear ITS, Mitochondrial DNA, Phylogeny

## Abstract

**Background:**

Despite the public health importance of toxocariasis/toxascariasis, only a few species of these ascaridoid parasites from wild canine and feline carnivores have been studied at the molecular level so far. Poor understanding of diversity, host distribution and the potential (zoonotic) transmission of the ascaridoid species among wild animals negatively affects their surveillance and control in natural settings. In this study, we updated previous knowledge by profiling the genetic diversity and phylogenetic relationships of ascaridoid species among eleven wild canine and feline animals on the basis of a combined analysis of the ribosomal internal transcribed spacer region (ITS) gene and the partial mitochondrial cytochrome *c* oxidase subunit 2 (*cox*2) and NADH dehydrogenase subunit 1 (*nad*1) genes.

**Results:**

In total, three genetically distinct ascaridoid lineages were determined to be present among these wild carnivores sampled, including *Toxocara canis* in *Alopex lagopus* and *Vulpes vulpes*, *Toxocara cati* in *Felis chaus*, *Prionailurus bengalensis* and *Catopuma temmincki* and *Toxascaris leonina* in *Canis lupus*, *Panthera tigris altaica*, *Panthera tigris amoyensis*, *Panthera tigris tigris*, *Panthera leo* and *Lynx lynx*. Furthermore, it was evident that *T. leonina* lineage split into three well-supported subclades depending on their host species, i.e. wild felids, dogs and wolves and foxes, based on integrated genetic and phylogenetic evidence, supporting that a complex of *T. leonina* other than one species infecting these hosts.

**Conclusions:**

These results provide new molecular insights into classification, phylogenetic relationships and epidemiological importance of ascaridoids from wild canids and felids and also highlight the complex of the taxonomy and genetics of *Toxascaris* in their wild and domestic carnivorous hosts.
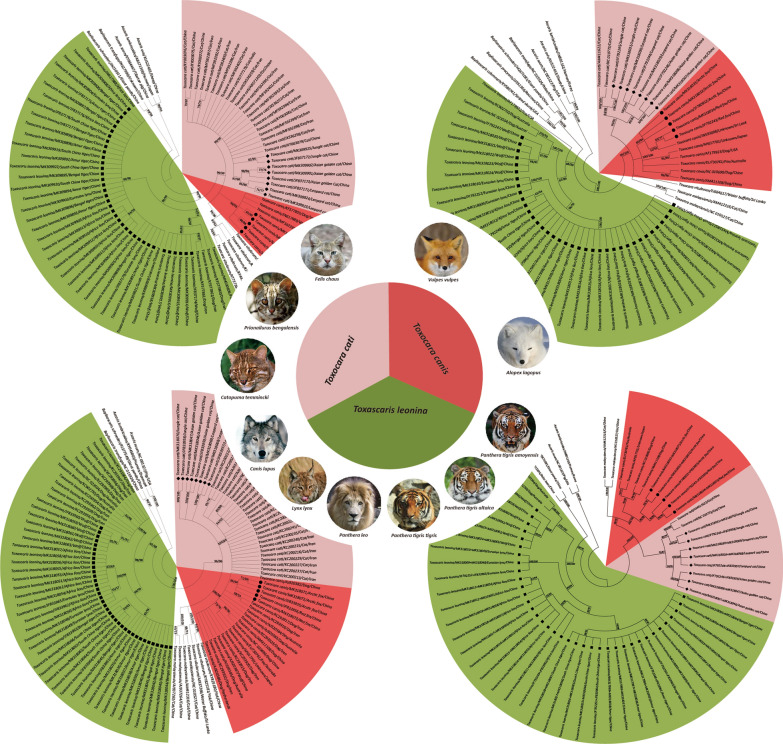

## Introduction

Ascaridoid nematodes of the genera *Toxocara* (Toxocaridae) and *Toxascaris* (Ascarididae) are the most common intestinal parasites among carnivores of the families Canidae and Felidae [1, 2]. Several species among them, such as *Toxocara canis* Werner, 1782, *T. cati* Schrank, 1788, *T. malaysiensis* Gibbons et al., 2001 and *Toxascaris leonina* Linstow, 1902 are regarded as zoonotic or potentially zoonotic parasites and pose threats to public health [3–8]. These parasites (at the adult stage) usually live in the small intestines of domestic and/or wild definitive hosts and cause significant health problems. The clinical symptoms include diarrhea, emesis, stunted growth, abdominal discomfort, and even life-threatening blockage of intestinal obstruction [9]. The definitive hosts of *T. canis* are dogs (*Canis familiaris*), dingoes (*Canis dingo*), wolfs (*Canis lupus*), coyotes (*Canis latrans*), jackals (*Canis aureus*), red foxes (*Vulpes vulpes*), arctic foxes (*Vulpes lagopus*), fennecs (*Megalotis zerda*) and a few feline species, while the definitive hosts for *T. cati* include cats (*Felis catus*), wild cats (*Felis silvestris*), servals (*Felis serwal*), cheetahs (*Actinomyx jubatus*), lynxes (*Lynx lynx*), pumas (*Puma concolor*), lions (*Panthera leo*), American leopards (*Panthera onca*), tigers (*Panthera tigris*), ocelots (*Leopardus pardalis*) and other felines [1]. Like *T. cati*, the congenetic *T. malaysiensis* infects felids [10]. However, *T. leonina* is defined to infect both feline and canine species [11, 12]. Humans are accidental hosts for *Toxocara* spp. and become infected through ingestion of infective eggs from the environment or articles contaminated with infected animal feces, with clinical forms including visceral larva migrans (VLM), ocular larva migrans (OLM), eosinophilic meningoencephalitis (EME), covert toxocariasis (CT) and neurological toxocariasis (NT) [2, 3].

Traditional approaches for the specific identification of ascaridoids within the genera *Toxocara* and *Toxascaris* rely on morphological characteristics, host preference and geographical distributions. However, these criteria are not always sufficient to distinguish the closely related and/or morphologically similar ascaridoid species, particularly when the larval and egg stages are involved [1, 12, 13]. Moreover, the diversity patterns of life-cycles (e.g. *Toxocara*) may complicate this procedure [4]. In such context, obtaining a more efficient and reliable approach to species identification has become crucial for clinical diagnosis and epidemiological investigation, and achieving this goal is foreseeable only through utilization of molecular methodologies [13]. Increased studies demonstrated that the nuclear ribosomal DNA (rDNA) and/or mitochondrial DNA (mtDNA) are valuable markers and have been often used to achieve the identification of parasites to species in *Toxocara* and *Toxascaris* [5, 6, 11, 12, 14–17]. For example, Jacobs et al. [18] used the second internal transcribed spacer sequence (ITS2) of rDNA to distinguish between *T. canis*, *T. cati* and *T. leonina* from foxes, cats and dogs in Australia. Likewise, based on the sequences of the internal transcribed spacers (ITS, ITS1 plus ITS2), Li et al. [15] developed a species-specific PCR tool for diagnosis of four ascaridoid species of dogs and cats including *T. canis*, *T. cati*, *T. malaysiensis* and *T. leonina* in China, Malaysia, Australia, England and the Netherlands. More recently, an ITS1-based PCR-RFLP was also employed for molecular determination of *T. cati* from wild felids in Argentina [19]. Compared to rDNA, however, mtDNA appears to be more variable both between and within species and is therefore more sensitive for genetic analysis of related ascaridoid species of *Toxocara* and *Toxascaris* [11, 16, 20]. In fact, the mitochondrial cytochrome *c* oxidase subunits 1 and 2 (*cox*1 and *cox*2) as well as NADH dehydrogenase subunits 1 and 4 (*nad*1 and *nad*4) genes have proven to be useful in resolving relationships among and between *Toxocara* spp. and *Toxascaris* spp. from dogs, cats and cattle [11, 12, 14, 16, 21, 22]; importantly, such mtDNA-derived evidence supports the rDNA-based conclusion that *T. malaysiensis* is another valid ascaridoid species found in cats [23]. Nevertheless, considering that single rDNA or mtDNA loci only allow limited inference of molecular analyses [24] and that current sampling and studies mostly focus on domestic animal-originated *Toxocara* and *Toxascaris* [4, 12], it would be essential and urgent to develop a combined analysis of nuclear and mitochondrial data for more accurate and robust branches in ascaridoids from a wider host-range that includes wild canids and felids. Unfortunately, until now there has been still very limited molecular information available for these ascaridoids [11, 12, 14, 25].

In the present study, ongoing epidemiological surveys on potential helminthic zoonoses, centred in the southwestern zoos of China, expanded previous investigations by including specimens of *Toxocara* and *Toxascaris* in 11 species of carnivores from Canidae and Felidae. Our aim was to characterise ascaridoids from an assemblage of diverse hosts relative to currently defined lineages of *Toxocara* and *Toxascaris* [1, 26], facilitating (i) identification of these isolates at species level by genetical analysis of the complete nuclear ITS and partial mitochondrial *cox*2 and *nad*1 markers; and (ii) determination of levels of genetic variation among these ascaridoids by comparisons among those documented in dogs and cats as well as other canids and felids which are available in public databases. This study should provide new molecular data for genetic diversity and phylogenetic relationships of ascaridoids from wild carnivorous animals and these results presented here also serve as molecular technical support for accurate diagnosis and better control of these parasites.

## Methods

### Sample collection and DNA extraction

**Fig. 1:**
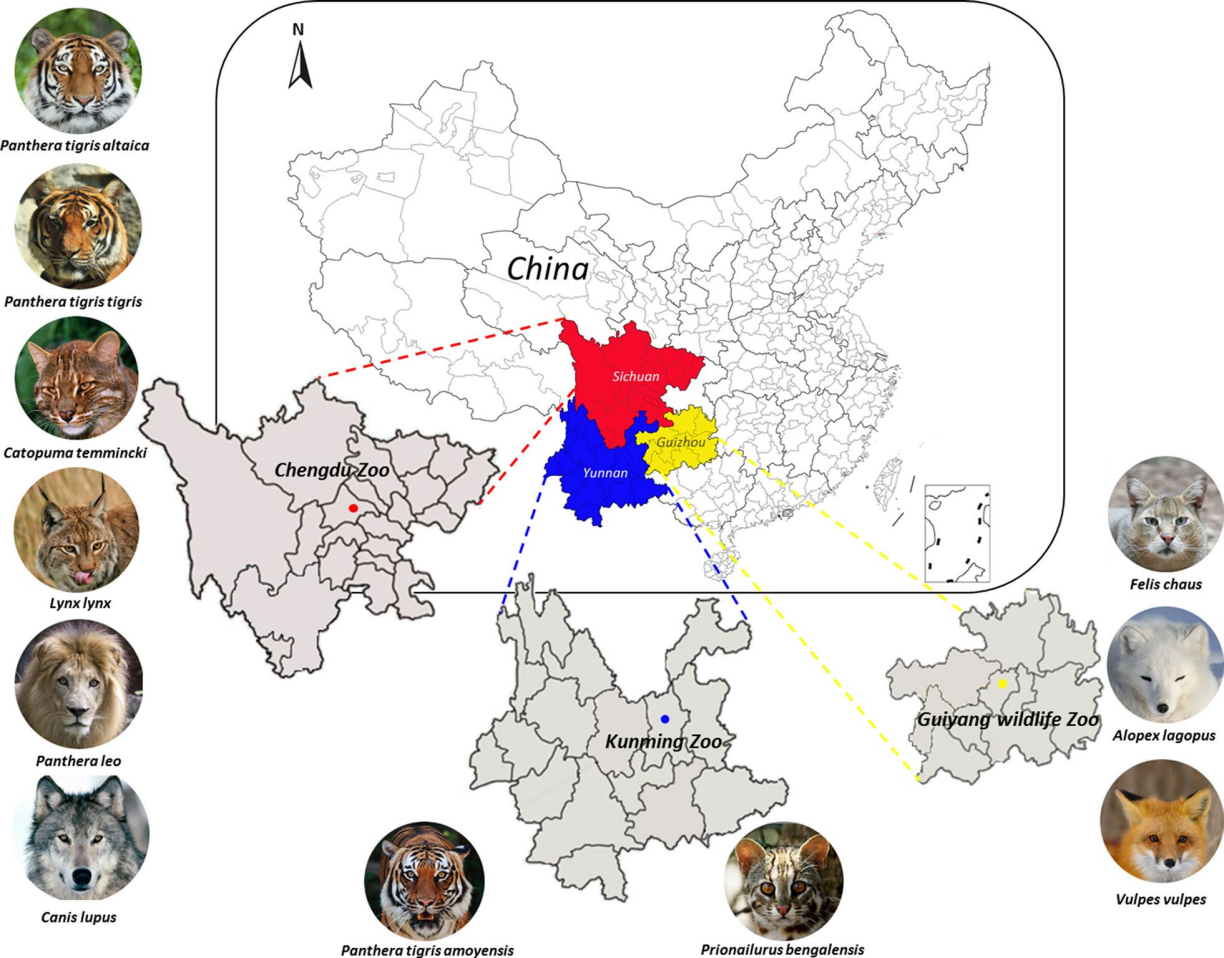
Sampling map in China showing the locations (coloured circles) of *Toxocara*/*Toxascaris* spp. collected from 11 wild canine and feline animal species housed in Chengdu Zoo of Sichuan Province (red), Kunming Zoo of Yunnan Province (blue) and Guiyang Wildlife Zoo of Guizhou Province (yellow)

During April 2011 to November 2018, a total of 50 adult ascaridoids were collected from 11 captive wild canine and feline animals after routine treatment with pyrantel pamoate, including the arctic fox (*Alopex lagopus*), red fox (*Vulpes vulpes*) and jungle cat (*Felis chaus*) in Guiyang Wildlife Zoo (Guizhou, China), South China tiger (*Panthera tigris amoyensis*) and leopard cat (*Prionailurus bengalensis*) in Kunming Zoo (Yunnan, China), and wolf (*Canis lupus*), Asian golden cat (*Catopuma temmincki*), Eurasian lynx (*Lynx lynx*), Bengal tiger (*Panthera tigris tigris*), Amur tiger (*Panthera tigris altaica*) and African lion (*Panthera leo*) in the Chengdu Zoo (Sichuan, China) (Table 1, Fig. 1). Each individual ascaridoid specimen was washed thoroughly in physiological saline solution and subjected to morphological identification according to taxonomic keys [27, 28]. Subsequently, all worm samples were stored at − 70 °C until required for DNA extraction. The genomic DNA (gDNA) of each isolate was extracted according to the method described by Jacobs et al. [18]. The DNA concentration and purity were measured by micro-spectrophotometry (NanoDrop ND-2000; Thermo Fisher Scientific, Wilmington, DE, USA).

**Table 1:** Summary information of canid and felid hosts and their ascaridoid nematodes sampled in this study

Host species	No. of sampled	
	Hosts	Parasite^a^
Wolf (*Canis lupus*)	3	6 (1/2/3)
Arctic fox (*Alopex lagopus*)	2	3 (1/2)
Red fox (*Vulpes vulpes*)	1	2
Asian golden cat (*Catopuma temmincki*)	2	3 (1/2)
South China tiger (*Panthera tigris amoyensis*)	3	5 (1/2/2)
Eurasian lynx (*Lynx lynx*)	2	3 (1/2)
African lion (*Panthera leo*)	3	10 (2/2/6)
Jungle cat (*Felis chaus*)	2	2 (1/1)
Amur tiger (*Panthera tigris altaica*)	1	6
Bengal tiger (*Panthera tigris tigris*)	2	7 (3/4)
Leopard cat (*Prionailurus bengalensis*)	3	3 (1/1/1)

^a^Parasite numbers sampled from each individual are shown in parentheses

### PCR amplification

The complete nuclear ITS sequence and partial sequences of the mitochondrial *cox*2 and *nad*1 were amplified by polymerase chain reaction (PCR) using the following primers: ITS (forward: 5′-GTA GGT GAA CCT GCG GAA GGA TCA TT-3′ and reverse: 5′-TTA GTT TCT TTT CCT CCG CT-3′); *cox*2 (forward: 5′-CAC CAA CTC TTA AAA TTA TC-3′ and reverse: 5′-TTT TCT AGT TAT ATA GAT TGR TTT YAT-3′) [22]; *nad*1 (forward: 5′-TTC TTA TGA GAT TGC TTT T-3′ and reverse: 5′-TAT CAT AAC GAA AAC GAG G-3′) [16]. All reactions were carried out in 50 μl reaction volume with 25 μl 2× *Taq* PCR MasterMix (Tiangen Biotech Co., Beijing, China), 3 μl gDNA, 16 μl sterile water and 3 μl of each primer (10 pmol/μl; TaKaRa Biotech, Dalian, China). The following cycle conditions were used in a Mastercycler Gradient 5331 Thermocycler (Eppendorf, Hamburg, Germany): 94 °C for 5 min; 35 cycles at 94 °C for 30 s, followed either by 60 °C for 30 s for ITS, 44 °C for 30 s for *cox*2 or 46 °C for 30 s for *nad*1, then 72 °C for 60 s; with a final extension step of 72 °C for 10 min. For each amplification, samples without parasite and host DNA were also included as negative controls to detect possible contamination. The PCR fragments were separated in 1% agarose gels, visualized using ethidium bromide and photographed by a Bio-Rad ChemiDoc XRS (Bio-Rad Laboratories, Hercules, CA, USA). The corrected gel-isolated amplicons were column-purified with the TIANgel Midi Purification Kit (Tiangen) and sub-cloned into *Escherichia coli* (DH5a) using the vector pMD-19T (TaKaRa). Positive clones were screened and were sequenced in-house on an ABI PRISMTM 377XL DAN sequencer (Invitrogen Biotechnology Co. Ltd., Shanghai, China). To ensure maximum accuracy, an individual clone from one specimen was sequenced four times independently using the M13F and M13R primers. The consensus sequences were deposited in the GenBank database under the accession numbers JF837169-JF837179 and MK309890-MK309928 (ITS), JF780952-JF792252 and MK317996-MK318034 (*cox*2) and JF833955-JF833965 and MK318035-MK318073 (*nad*1).

### Sequence analysis and phylogeny

The ITS, *cox*2 and *nad*1 sequences of ascaridoid species in the present study were initially edited with BioEdit (Ibis Biosciences, Carlsbad, USA) and subjected to separate sequence similarity search using BLAST (NCBI; http://blast.ncbi.nlm.nih.gov/Blast.cgi) to identify the nearest phylogenetic neighbours, and then aligned with ClustalX 1.83 [29]. All reference sequences from GenBank with parasite species, host species and geographical origins are listed in Table 2. During alignment, a codon-guided protein alignment was used for manual adjustment of the nucleotide alignments of *cox*2 and *nad*1. Pairwise comparisons of sequence identities (I) were calculated among and within species using the formula I = M/L, where M is the number of alignment positions at which the two sequences have a same base, and L is the total number of alignment positions included in the two sequences. Given almost identical nucleotide sequences of the ITS region and *cox*2 and *nad*1 genes in worms from the same host species in this study, one representative ascaridoid identified here was also selected and compared with *T. canis*, *T. cati*, *T. malaysiensis*, *T. vitulorum* and *T. leonina* as well as *Baylisascaris* spp. and *Ascaris* spp. for further detection of synonymous and non-synonymous mutations in the mitochondrial *cox*2 and *nad*1 genes according to their corresponding amino acid alignments, followed by calculation of genetic distances between them using a distance matrix based on the Kimura 2-parameter (K2P) model in MEGA 6.1 [30]. For phylogenetic analyses, two different methods, namely Bayesian inference (BI) (MrBayes 3.1.2 [31]) and maximum likelihood (ML) (PHYML 3.0 [32]) were carried out based on the refined sequence alignment datasets by the online Gblocks server (http://molevol.cmima.csic.es/castresana/Gblocks_server.html). For the BI analysis, the general time reversible (GTR) including gamma-distributed rate variation (+G) and a proportion of invariable sites (+I) (GTR + G + I; ITS) and gamma-distributed rate variation (GTR + G; *cox*2, *nad*1 and *cox*2 and *nad*1) was determined as the best-fit nucleotide substitution model using the Bayesian information criteria (BIC) test in jModeltest 2.1.6 [33], and the trees were constructed using four independent Markov chain runs for 10,000,000 (ITS), 100,000 (*cox*1), 50,000 (*nad*1) or 10,000,000 (*cox*1 and *nad*1) metropolis-coupled MCMC generations with every 10,000th (ITS), 100th (*cox*1), 50th (*nad*1) or 10,000th (*cox*1 and *nad*1) tree being sampled; when the average standard deviation of the split frequencies dropped below 0.01, 25% initial trees were discarded as “burn-in” and the remaining trees were used to calculate posterior probabilities and visualised graphically with TreeviewX (http://darwin.zoology.gla.ac.uk/~rpage/treeviewx/). The ML computations were performed using PHYML 3.0 under the GTR model for ITS and GTR + I for *cox*2, *nad*1 and *cox*2 and *nad*1. Both *Baylisascaris* spp. and *Ascaris* spp. were used as reference outgroups in phylogenetic analyses. In addition, given recent studies showing a genetic separation among *T. leonina* depending on their host origins, these newly generated and previously published sequences of the nuclear and mitochondrial genes of *T. leonina* were also concatenated and used to test the assumption using the same phylogenetic methods as described above.

**Table 2:** Summary information of *Toxocara*/*Toxascaris* and other related ascaridoid species used for molecular identification in the present study

Parasites	Stages	Host species	Geographical origin			Living conditions	GenBank accession number			References
			*ITS*	*cox*2	*nad*1		*ITS*	*cox*2	*nad*1	
*Toxascaris leonina*	Adult	South China tiger	China	China	China	Zoo	JF837178	JF792251	JF833964	This study
	Adult	African lion	China	China	China	Zoo	JF837176	JF792249	JF833962	This study
	Adult	Wolf	China	China	China	Zoo	JF837174	JF792247	JF833960	This study
	Adult	Eurasian lynx	China	China	China	Zoo	JF837179	JF792252	JF833965	This study
	Adult	Bengal tiger	China	China	China	Zoo	JF837177	JF792250	JF833963	This study
	Adult	Amur tiger	China	China	China	Zoo	JF837175	JF792248	JF833961	This study
	Adult	Wolf	China	China	China	Zoo	MK309918	MK318024	MK318063	This study
	Adult	Wolf	China	China	China	Zoo	MK309917	MK318023	MK318062	This study
	Adult	Wolf	China	China	China	Zoo	MK309916	MK318022	MK318061	This study
	Adult	Wolf	China	China	China	Zoo	MK309915	MK318021	MK318060	This study
	Adult	Wolf	China	China	China	Zoo	MK309914	MK318020	MK318059	This study
	Adult	Amur tiger	China	China	China	Zoo	MK309890	MK317996	MK318035	This study
	Adult	Amur tiger	China	China	China	Zoo	MK309891	MK317997	MK318036	This study
	Adult	Amur tiger	China	China	China	Zoo	MK309892	MK317998	MK318037	This study
	Adult	Amur tiger	China	China	China	Zoo	MK309893	MK317999	MK318038	This study
	Adult	Amur tiger	China	China	China	Zoo	MK309894	MK318000	MK318039	This study
	Adult	African lion	China	China	China	Zoo	MK309905	MK318011	MK318050	This study
	Adult	African lion	China	China	China	Zoo	MK309913	MK318019	MK318058	This study
	Adult	African lion	China	China	China	Zoo	MK309912	MK318018	MK318057	This study
	Adult	African lion	China	China	China	Zoo	MK309911	MK318017	MK318056	This study
	Adult	African lion	China	China	China	Zoo	MK309910	MK318016	MK318055	This study
	Adult	African lion	China	China	China	Zoo	MK309907	MK318013	MK318052	This study
	Adult	African lion	China	China	China	Zoo	MK309906	MK318012	MK318051	This study
	Adult	Eurasian lynx	China	China	China	Zoo	MK309904	MK318010	MK318049	This study
	Adult	African lion	China	China	China	Zoo	MK309908	MK318014	MK318053	This study
	Adult	African lion	China	China	China	Zoo	MK309909	MK318015	MK318054	This study
	Adult	Bengal tiger	China	China	China	Zoo	MK309895	MK318001	MK318040	This study
	Adult	Bengal tiger	China	China	China	Zoo	MK309896	MK318002	MK318041	This study
	Adult	Bengal tiger	China	China	China	Zoo	MK309897	MK318003	MK318042	This study
	Adult	Bengal tiger	China	China	China	Zoo	MK309898	MK318004	MK318043	This study
	Adult	Bengal tiger	China	China	China	Zoo	MK309899	MK318005	MK318044	This study
	Adult	Bengal tiger	China	China	China	Zoo	MK309900	MK318006	MK318045	This study
	Adult	South China tiger	China	China	China	Zoo	MK309919	MK318025	MK318064	This study
	Adult	South China tiger	China	China	China	Zoo	MK309920	MK318026	MK318065	This study
	Adult	South China tiger	China	China	China	Zoo	MK309921	MK318027	MK318066	This study
	Adult	Eurasian lynx	China	China	China	Zoo	MK309903	MK318009	MK318048	This study
	Adult	South China tiger	China	China	China	Zoo	MK309922	MK318028	MK318067	This study
	Adult	African lion	China	China	China	Zoo	JN617988	–	–	Unpublished
	Adult	Red fox	USA	USA	–	–	–	AF179922	–	Nadler and Hudspeth [22]
	Adult	Dog	–	Australia	Australia	Domestication	–	NC_023504	NC_023504	Liu et al. [35]
	Adult	Dog	Iran	–	–	Domestication	KF577860-62	–	–	Unpublished
	Adult	Fox	USA	–	–	Zoo	MH030606	–	–	Hoberg et al. [67]
	Adult	Unknown	Thailand	–	–	–	KR999999	–	–	Unpublished
	Adult	Dog	–	–	Iran	In the wild	–	–	KC293969, KC293947-69	Fogt-Wyrwas et al. [12]
	Adult	Fox	Poland	–	–	In the wild	HM800922	–	–	Fogt-Wyrwas et al. [12]
	Adult	Dog	Iran	–	–	Domestication	KF577860-62	–	–	Fogt-Wyrwas et al. [12]
*Toxocara cati*	Adult	Jungle cat	China	China	China	Zoo	JF837172	JF792245	JF833958	This study
	Adult	Asian golden cat	China	China	China	Zoo	JF837173	JF792246	JF833959	This study
	Adult	Leopard cat	China	China	China	Zoo	JF837171	JF792244	JF833957	This study
	Adult	Jungle cat	China	China	China	Zoo	MK309925	MK318031	MK318070	This study
	Adult	Asian golden cat	China	China	China	Zoo	MK309902	MK318008	MK318047	This study
	Adult	Asian golden cat	China	China	China	Zoo	MK309901	MK318007	MK318046	This study
	Adult	Leopard cat	China	China	China	Zoo	MK309924	MK318030	MK318069	This study
	Adult	Leopard cat	China	China	China	Zoo	MK309923	MK318029	MK318068	This study
	Adult	Cat	Japan			Domestication	AB571303	–	–	Arizono et al. [68]
	Adult	Cat	–	China	China	In the wild	–	NC_010773	NC_010773	Li et al. [69]
	Adult	Cat	China	–	–	Domestication	KY003072, KY003075-76, KY003079-81	–	–	He et al. [70]
	Egg	Cat	Iran	–	–	In the wild	MF592392-99, MF592400-02	–	–	Unpublished
	Adult	Cat	Iran	–	–	In the wild	JX536257-59	–	–	Unpublished
	Adult	Cat	India	–	–	Domestication	KJ777179	–	–	Unpublished
	Adult	Cat	–	–	Iran	Domestication	–	–	KC200213-17, KC200222-23, KC200225-27, KC200229-32, KC200235, KC200237, KC200239-41, KC200247	Mikaeili et al. [21]
	Egg	Cat	–	–	Iran	Domestication	–	–	KC200246	Mikaeili et al. [21]
	Adult	Cat	–	–	UK	Domestication	–	–	AJ937261	Li et al. [16]
	Adult	Cat	–	China	China	Domestication	–	AM411622	AM411622	Li et al. [69]
	Adult	Cat	–	–	Australia	–	–	–	AJ937262	Li et al. [16]
*Toxocara canis*	Adult	Red fox	China	China	China	Zoo	JF837170	JF792243	JF833956	This study
	Adult	Arctic fox	China	China	China	Zoo	JF837169	JF780952	JF833955	This study
	Adult	Red fox	China	–	–	Zoo	MK309928	MK318034	MK318073	This study
	Adult	Arctic fox	China	–	–	Zoo	MK309926	MK318032	MK318071	This study
	Adult	Arctic fox	China	–	–	Zoo	MK309927	MK318033	MK318072	This study
	Adult	Fox	–	Australia	Australia	In the wild	–	EU730761	EU730761	Jex et al. [71]
	Unknown	Unknown	–	Japan	Japan	Unknown	–	AP017701	AP017701	Unpublished
	Unknown	Unknown	–	Sri Lanka	–	Unknown	–	JN593098	–	Wickramasinghe et al. [72]
	Adult	Wolf	China	–	–	Zoo	JN617989	–	–	Unpublished
	Adult	Dog	–	USA	–	–	–	AF179923	–	Nadler and Hudspeth [22]
	Adult	Dog	–	China	China	Domestication	–	NC_010690	NC_010690	Li et al. [69]
	Egg	Dog	Iran	–	–	In the wild	MF592391	–	–	Choobineh et al. [73]
	Adult	Dog	Iran	–	–	In the wild	KF577855	–	–	Unpublished
	Adult	Dog	–	–	Iran	In the wild	–	–	KC293915-17, KC293920-23	Mikaeili et al. [21]
	Adult	Dog	–	–	Australia	In the wild	–	–	AJ920383-85	Li et al. [16]
	Adult	Dog	–	–	China	In the wild	–	–	AJ920382	Li et al. [16]
	Larvae	Dog	–	–	Netherlands	–	–	–	AJ920386	Li et al. [16]
	Adult	Cat	–	–	China	Domestication	–	–	AJ920387	Li et al. [16]
*Toxocara malaysiensis*	Adult	Cat	–	China	China	Domestication	–	AM412316	AM412316	Li et al. [16]
	Adult	Cat	China	–	–	Domestication	AM231609	–	–	Li et al. [16]
	Adult	Cat	–		China	Domestication	–	–	AJ937263-65	Li et al. [16]
*Toxocara vitulorum*	Adult	Yak	India	–	–	Domestication	KJ777180	–	–	Unpublished
	Adult	Cattle	India	–	–	–	KJ777181	–	–	Unpublished
	Adult	Mithun calf	India	–	–	–	KJ777182	–	–	Unpublished
	Adult	Cattle	USA	–	–	Domestication	KT737382	–	–	Unpublished
	Adult	European bison	Germany	–	–	–	KY442062	–	–	Unpublished
	Egg	Water buffalo	–	–	Sri Lanka	–	–	–	AJ937266	Li et al. [16]
	Adult	Yak	–	–	China	Domestication	–	–	KY825180-81	Li et al. [74]
	Adult	Water buffalo	–	Sri Lanka	–	Domestication	–	FJ664617	–	Wickramasinghe et al. [75]
*Baylisascaris transfuga*	Adult	Unknown	China	–	–	Zoo	JN617990	–	–	Unpublished
	Adult	Polar bear	–	China	China	Zoo	–	NC_015924	NC_015924	Xie et al. [37]
*Baylisascaris columnaris*	Adult	Striped skunk	–	USA		In the wild	–	KY580741	–	Choi et al. [76]
*Baylisascaris procyonis*	Adult	Raccoon	–	China	China	Zoo	–	JF951366	JF951366	Xie et al. [33]
*Baylisascaris schroederi*	Adult	Giant panda	–	China	China	Zoo	–	HQ671081	HQ671081	Xie et al. [37]
	Adult	Giant panda	–	–	China	Zoo	–	–	FJ377549	Unpublished
	Adult	Giant panda	China	–		Zoo	JN210911	–	–	Lin et al. [77]
*Ascaris ovis*	Adult	Sheep	–	China	China	–	–	KU522453	KU522453	Unpublished
	Adult	Sheep	China	–	–	–	KU522455	–	–	Unpublished
*Ascaris suum*	Adult	Tibetan pig	China	–	–	Domestication	KY964447	–	–	Li et al. [78]
	Adult	Pig	–	USA	USA	Domestication	–	NC_001327	NC_001327	Wolstenholme et al. [79]
*Ascaris lumbricoides*	Adult	Human	–	Korea	Korea	Domestication	–	JN801161	JN801161	Park et al. [80]
	Adult	Human	–	Denmark	Denmark	Domestication	–	KY045803	KY045803	Unpublished
	Adult	Human	Japan	–	–	Domestication	AB571300	–	–	Arizono et al. [68]

## Results

### Sequence characterization

The DNA sequences representing the ITS (856–975 bp) region and *cox*2 (582 bp) and *nad*1 (366 bp) genes were generated for all 50 ascaridoid isolates. Sequence alignments showed common insertions/deletions (indels) in the ITS region with 314 variable sites including 313 parsimony-informative and one singleton sites; while compared to the ITS, both *cox*2 and *nad*1 genes appeared more conserved in either length or base composition with 135 parsimony-informative sites for *cox*2 and 93 variable sites for *nad*1 (no singleton sites for both genes; Additional file 1: Table S1). The mean A + T contents of *cox*2 and *nad*1 were 66.5% and 68.6%, respectively, a typical mitochondrial nucleotide feature in nematodes (AT bias). BLAST searches using either ITS, *cox*2 or *nad*1 sequence all assigned the 50 ascaridoid isolates into two groups, and one group had highest nucleotide identities to representative reference sequences for specimens of *Toxocara* (97.6–99.6% for ITS, 93.0–99.8% for *cox*2 and 91.3–98.6% for *nad*1) and another had highest nucleotide identities to representative reference sequences for specimens of *Toxascaris* (100% for ITS, 98.5% for *cox*2 and 98.9% for *nad*1). Within the *Toxocara* group, 5 of 13 ascaridoids shared the highest nucleotide identity with *T. canis* (99.6–99.9%; GenBank accession numbers JN617989 for ITS, JN593098 for *cox*2 and KC293917 for *nad*1) and the remaining 8 ascaridoids exhibited high nucleotide identities with *T. cati* (89.3–94.5%; GenBank accession numbers KY003079 for ITS, AM411622 for *cox*2 and KC200223 for *nad*1). For the *Toxascaris* group, all 37 ascaridoids showed high nucleotide identities with *T. leonina* (90.7–96.8%; GenBank accession numbers KR999999 for ITS, KC902750 for *cox*2 and KC293956 for *nad*1). Following the identity comparisons, the conserved and genus-specific nucleotide sites of ITS, *cox*2 and *nad*1were also identified by adding the congeneric species *T. canis*, *T. cati*, *T. malaysiensis* and *T. vitulorum* as well as *T. leonina* and other related species, *Baylisascaris* spp. and *Ascaris* spp. It was evident that both *cox*2 and *nad*1 exhibited more-stringent nucleotide sequence conservation than that of ITS. Thus, we mainly focused on the conserved sites in these two mitochondrial genes and detected their variable sites in the same regions as well, in order to determine if the base conservations were *Toxocara* and *Toxascaris*-specific and if there were non-synonymous substitutions apparent in these two genes by respective comparisons of their protein sequences in representative specimens. As shown in Additional file 2: Figure S1 and Additional file 3: Figure S2, we found that among the conserved base sites of *cox*2 9 were *Toxocara*-specific (50:G, 215:A, 376:A, 377:C, 439:G, 507:T, 563:T, 577:T and 579:G) and 3 were *Toxascaris*-specific (271:G, 273:A and 498:A); likewise, among the conserved base sites of *nad*1 3 were *Toxocara*-specific (71:C, 273:T and 276:G) and 3 were *Toxascaris*-specific (62:A, 262:G and 263:T). Among the variable base sites of *cox*2 and *nad*1, however, 13 (*cox*2) and 8 (*nad*1) were found to be unique for *Toxocara* spp. (in red), and 3 (*cox*2) and 5 (*nad*1) were unique for *Toxascaris* spp. (in blue). Importantly, among these variable sites 6 were confirmed to be non-synonymous substitutions based on respective protein alignments of *cox*2 and *nad*1, which lead to a total of 6 amino-acid changes, including 25:K/R/S(Val/Arg/Ser) → C(Ilu), 135:G(Gly) → S(Ser) and 167:V/I(Val/Ilu) → L(Leu) in *cox*2 and 25:V/F(Val/Phe) → I(Ilu), 89:L(Leu) → M(Met) and 104:L/F/I/C(Leu/Phe/Ilu/Cys) → V(Val) in *nad*1 (see Additional file 2: Figure S1, Additional file 3: Figure S2).

### Evolutionary distance analysis

The evolutionary distances among the 11 representative specimens of *Toxocara*/*Toxascaris* and with other closely related ascaridoids were estimated and are shown in Additional file 4: Table S2 and Additional file 5: Table S3. Our analysis showed that among these *Toxocara*/*Toxascaris* isolates their evolutionary distances varied depending on the different genetic markers used. For example, the ascaridoid species in the Asian golden cat showed a minimum intraspecific evolutionary distance (0.036) with that in the jungle cat in ITS-based analysis while the value changed into 0.108 in the *cox*2 and 0.124 in the *nad*1 data. Nevertheless, the three gene datasets consistently placed ascaridoid species in either the arctic fox or red fox close to *T. canis* in the domestic dog, ascaridoid species in either the jungle cat, leopard cat or Asian golden cat close to *T. cati* in the domestic cat, and ascaridoid species in either the wolf, South China tiger, Eurasian lynx, African lion, Amur tiger or Bengal tiger close to *T. leonina* in the domestic dog or wild fox with the minimum intraspecific evolutionary distances of 0.002–0.004 for ITS, 0.003 for *cox*2 and 0.007–0.010 for *nad*1, 0.006–0.010 for ITS, 0.008–0.012 for *cox*2 and 0.003–0.017 for *nad*1, and 0.000–0.003 for ITS, 0.007–0.010 for *cox*2 and 0.003–0.010 for *nad*1, respectively, in accordance with conclusions of our identity analysis. More significant divergence was found in comparisons to *T. vitulorum* with 0.096–0.264 (ITS), 0.093–0.131 (*cox*2) and 0.127–0.193 (*nad*1), *Ascaris* spp. with 0.192–0.303 (ITS), 0.091–0.149 (*cox*1) and 0.153–0.181 (*nad*1) and *Baylisascaris* spp. with 0.190–0.307 (ITS), 0.097–0.173 (*cox*1) and 0.123–0.185 (*nad*1) (see Additional file 4: Table S4, Additional file 5: Table S3).

### Phylogenetic analysis

**Fig. 2:**
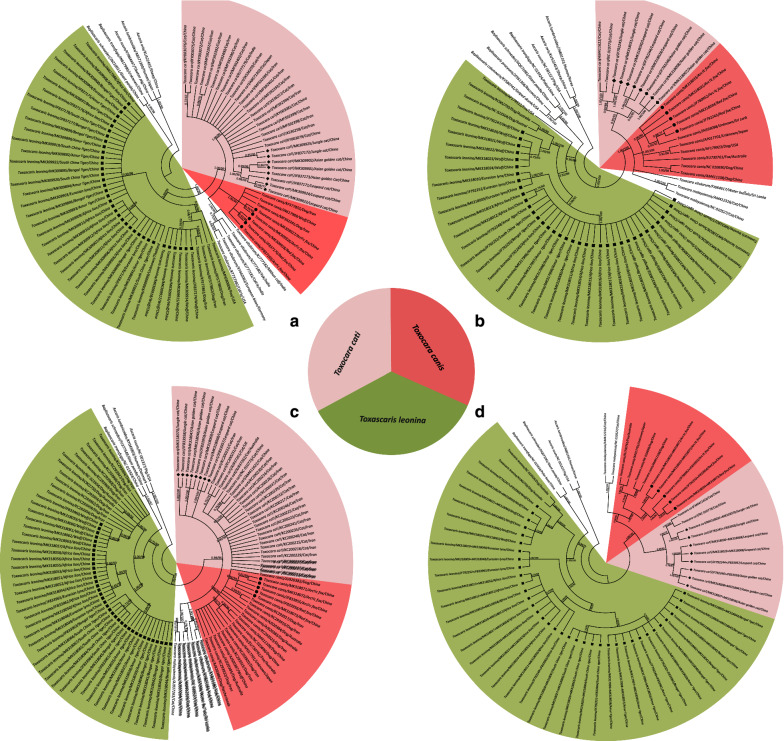
Phylogenetic relationships of *Toxocara*/*Toxascaris* spp. from wild and domestic canine and feline carnivores and other related ascaridoid species. The phylogenetic topologies were inferred on the basis of nuclear ITS (**a**) and mitochondrial *cox*2 (**b**) and *nad*1 (**c**) as well as a combination of *cox*2 and *nad*1 (**d**) sequences using Bayesian inference (BI) and maximum likelihood (ML) methods. The reference sequences for species of *Ascaris* were used the outgroups. All species information about ascaridoid nematodes included in this study is summarized in Table 1. Species of *Toxocara*/*Toxascaris* recovered from wild and domestic canids and felids were genetically divided into three lineages, i.e. *Toxocara canis*, *T. cati* and *Toxascaris leonina* and indicated by three differently coloured sectors with *T. canis* in red, *T. cati* in pink and *T. leonina* in green. The numbers along the branches show bootstrap values resulting from different analyses in the order BI/ML; values < 50% are not shown

**Fig. 3:**
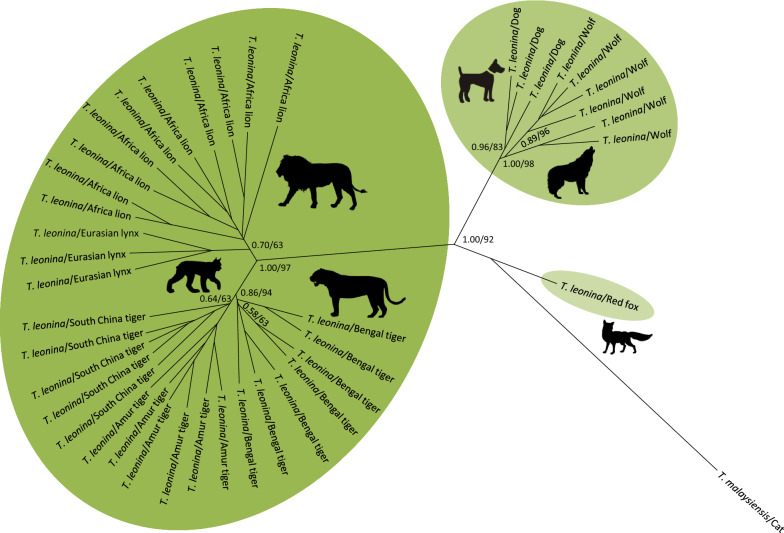
Inferred phylogenetic relationships among *Toxascaris leonina* isolates from different hosts by Bayesian inference (BI) and maximum likelihood (ML) methods based on a combination of the nuclear (ITS) and mitochondrial (*cox*2 + *nad*1) nucleotide sequence data, using *Toxocara malaysiensis* as the outgroup. The numbers along the branches show bootstrap values resulting from different analyses in the order BI/ML; values < 50% are not shown

The phylogeny of the 50 specimens of *Toxocara*/*Toxascaris* and their relationships with other related ascaridoid species were inferred on the basis of the respective sequences of ITS, *cox*2 and *nad*1 as well as a combination of *cox*2 and *nad*1 using both BI and ML methods, and their corresponding tree topologies are showed in Fig. 2. Although four identical trees (BI/ML) topologically differed from each other because of genes and reference species included here, all analyses yielded a consistent, robust phylogenetic resolution for these 50 ascaridoid isolates and their congeneric species in the genera *Toxocara* in the family Toxocaridae and *Toxascaris* in the family Ascarididae. In total, three unequivocal clades including all ascaridoid isolates identified here were demonstrated, suggesting varying patterns of broad to relatively narrow host range. Among them, one clade placed the ascaridoid isolates of the arctic fox and red fox together with the dog/wolf/fox *T. canis* and showed a sister relationship with another clade that contained the ascaridoid isolates of the jungle cat, leopard cat and Asian golden cat as well as the cat *T. cati*, with almost maximum support values for three tree topologies (bootstrap values: 1.00/99/, 1.00/100, 0.98/96 or 1.00/100); we named these two clades as *T. canis* (red sectors) and *T. cati* (pink sectors) lineages, respectively (Fig. 2). In other words, the ascaridoids from the arctic fox and red fox were identified as *T. canis* and the ascaridoids from the jungle cat, leopard cat and Asian golden cat were identified as *T. cati* in this study. Combined with the congeneric *T. malaysiensis* and *T. vitulorum* lineages, these species showed a sister-group relationship in the genus *Toxocara* within Toxocaridae. Within the third clade, although the ascaridoid isolates of the wolf, South China tiger, Eurasian lynx, African lion, Amur tiger and Bengal tiger clustered with the dog/fox *T. leonina* with high statistical supports (bootstrap values: 1.00/99, 0.90/88, 1.00/98 or 1.00/100) and were referred as *T. leonina* lineage (green sectors), this lineage was more closely related to species of the Toxocaridae than other species of the Ascarididae in both ITS- and *cox*2-based analyses (Fig. 2a, b), in contrast with a close relationship between *T. leonina* and *Baylisascaris* spp. based on *nad*1 and *cox*2 plus *nad*1 analyses (Fig. 2c, d). Moreover, within the *T. leonina* lineage, it was notable that these *T. leonina* representatives appeared to belong to three distinct subclades depending to their host species, i.e. *T. leonina* from wild felids (such as lions, tigers and lynxes) in one subclade and *T. leonina* from canid hosts in another two subclades, including the dog/wolf-*T. leonina* subclade and fox-*T. leonina* subclade. Similar phylogenetic relationships were also supported when using a combination of the nuclear and mitochondrial data of *T. leonina* (Fig. 3), suggesting a cryptic speciation of *T. leonina*. For the inter-relationships of *Baylisascaris* spp. and *Ascari*s spp. and both with *Toxocara* spp. including *T. canis*, *T. cati*, *T. malaysiensis* and *T. vitulorum*, the phylogenetic topologies were in agreement with previously proposed molecular phylogenies of the ascaridoids based on nuclear and mitochondrial DNA data [33–37], confirming the phylogenetic stability of these paraphyletic groups characterised in this study.

## Discussion

*Toxocara* and *Toxascaris* are the most common intestinal parasites of domestic and wild canids and felids and can cause human toxocariasis (mainly involving the species of *Toxocara*) with an important public health impact [1–4, 7, 8]. Although morphological characteristics and host preferences can be used for species identification, these existing keys and descriptions are often insufficient particularly when differentiation is required within and between *Toxocara* spp. and *Toxascaris* spp. and especially their larval and egg stages [1, 12, 13]. Similar problems may be exacerbated in attempts to perform species-specific identification among certain wild animal-derived species. Species of *Toxocara* and *Toxascaris* in wild canids and felids are considered not only because these wildlife hosts may have recently adapted to the human-environment due to rapid urbanization leading to increased interactions with people in conservation centers and zoological gardens, but also because little attention has been paid to these ascaridoids due to the limited access to samples [2, 38–41]. Such situations would negatively affect their diagnosis and surveillance. Recent advances in molecular diagnosis provide substantial opportunities for overcoming this problem. Polymerase chain reaction (PCR)-based approaches using genetic markers from rDNA and/or mtDNA have proven to be effective and have been used for large-scale studies on the identification and differentiation of ascaridoids to the species level [5, 6, 12, 15–17]. In this study, the ascaridoids from 11 captive wild canine and feline hosts were identified and characterised by PCR amplifying and genetically analyzing the nuclear ITS and the partial mitochondrial *cox*2 and *nad*1 genes.

Fifty ascaridoid isolates in this study were morphologically identified as *Toxocara* (*n* = 13) or *Toxascaris* (*n* = 37) according to the presence of a post-oesophageal bulbus and the length and shape of the cervical alae [26–28]. Further pairwise comparisons of ITS showed that among the 13 *Toxocara* isolates the ascaridoids in the arctic fox and red fox were found to share high nucleotide identities with representative references from specimens of *T. canis*, while the ascaridoids in the jungle cat, leopard cat and Asian golden cat had high nucleotide identities to representative references from specimens of *T. cati* in the cat. For the 37 *Toxascaris* isolates that were in the wolf, South China tiger, Eurasian lynx, African lion, Amur tiger and Bengal tiger, ITS identity analysis showed their high nucleotide identities with representative references from specimens of *T. leonina*. Thus, it can be assumed that there may be three ascaridoid species representing *T. canis*, *T. cati* and *T. leonina* among the ascaridoid isolates studied here. To confirm this assumption, ITS-based phylogenetic analyses (BI/ML) were performed and the results once again showed that the ascaridoids in the arctic fox and red fox grouped with *T. canis*, the ascaridoids in the jungle cat, leopard cat and Asian golden cat grouped with *T. cati*, and the ascaridoids in the wolf, South China tiger, Eurasian lynx, African lion, Amur tiger and Bengal tiger grouped with *T. leonina*, with high bootstrap values (see Fig. 2a), supporting that *T. canis* and *T. leonina* can co-occur in wild canids and *T. cati* and *T. leonina* can co-occur in wild felids [42–46]. Encouragingly, this conclusion was strengthened by analyses of mitochondrial *cox*2 and *nad*1. Both *cox*2 and *nad*1 were included because of their utility in identifying and differentiating nematode species among very closely related taxa due to their fast mutation rates, maternal inheritance and lack of recombination [16, 47, 48]. Similar to the nuclear ITS, high nucleotide identities of *cox*2 and *nad*1 were also observed between *T. canis* and the ascaridoids in either the arctic fox and red fox, between *T. cati* and the ascaridoids in either the jungle cat, leopard cat and Asian golden cat, and between *T. leonina* and the ascaridoids in either the wolf, South China tiger, Eurasian lynx, African lion, Amur tiger and Bengal tiger (see Additional file 2: Figure S1, Additional file 3: Figure S2). Importantly, consistent strong bootstrap support was evident, on the basis of phylogenetic analyses (MP/ML) of *cox*2 and *nad*1 that verified the same species of *Toxocara* in either the arctic fox and red fox (i.e. *T. canis*) or the jungle cat, leopard cat and Asian golden cat (i.e. *T. cati*), and the same species of *Toxascaris* in the wolf, South China tiger, Eurasian lynx, African lion, Amur tiger and Bengal tiger (i.e. *T. leonina*) (see Fig. 2b, d). Of course, possible cross-infection could be a confounder for this outcome because of the close or sympatric housing conditions for these captive wild canids and felids. This possibility was refuted because sampling records, particularly for the three species of the Felinae, showed that the jungle cat was housed in the Guiyang Wildlife Zoo (Guizhou, China) and the leopard cat in the Kunming Zoo (Yunnan, China), while the Asian golden cat was kept in the Chengdu Zoo (Sichuan, China). Besides, the captive history also showed that these host species were bred in zoos and no translocations and/or introductions occurred before, suggesting that these *Toxocara*/*Toxascaris* spp. were present with the original captive populations of these wild canids and felids and their own infection cycles may have been maintained over time.

Building on the results from integrated molecular evidence, we propose that *Toxocara* spp. from either wild canids or felids represent the same species but belong to different lineages in the genus *Toxocara*, i.e. *Toxocara* sp. of the arctic fox and red fox was within the *T. canis* lineage and *Toxocara* sp. of the jungle cat, leopard cat and Asian golden cat was within the *T. cati* lineage. In fact, since Jacobs et al. [18] first demonstrated the ascaridoid of the fox as *T. canis*, increased epidemiological studies have produced a consistent conclusion that *T. canis* is the common ascaridoid of foxes, regardless of fox species and originations [1, 49–52]. For instance, *T. canis* infections in red foxes have been reported in Great Britain (estimated prevalence: 62%) [45], Denmark (49%) [51], Switzerland (44%) [53], Norway (3.5–41.2%) [50], Ireland (20%) [54], Poland (11%) [55] and Italy (9%) [49]. Moreover, Meijer et al. [56] showed a prevalence of 7% and 30% of *T. canis* in arctic foxes during two summers (2008 and 2010, respectively) in Sweden. For *T. cati*, this parasite is commonly found in cats including wild species. Like *T. cati* in golden cats, jungle cats and leopard cats included here, *T. cati* infections have been reported in feral cats in Spain (35%) [57], stray cats in Iran (43%) [58] and wild cats in Egypt (59%) [59] and Italy (44%) [60]. Thus, taking into account the fact that canids and felids are natural hosts for *T. canis* and *T. cati*, respectively, and combining this with the aforementioned genetic evidence, our proposal that the same *Toxocara* species parasitizes either the arctic fox and red fox (*T. canis*) or the jungle cat, leopard cat and Asian golden cat (*T. cati*) is reasonable. Of course, additional information regarding ultrastructure and complete genomic information of these *Toxocara* species and other related ascaridoids as well as broader taxonomic comparisons is still required to provide an increasingly precise morphological and molecular basis for species recognition among ascaridoids.

In addition, our phylogenetic analyses placed *Toxascaris* sp. of the wolf, South China tiger, Eurasian lynx, African lion, Amur tiger and Bengal tiger into the *T. leonina* lineage (Fig. 2). It is clear that *T. leonina* is a cosmopolitan and polyxenical parasite in wild and domestic canids and felids [1]. However, recent studies by Fogt-Wyrwas et al. [12], Jin et al. [11] and Xie et al. [61] consistently pointed out that *T. leonina* may be a species complex. Because combined molecular evidence from ribosomal nuclear DNA strongly showed the separation of *T. leonina* from different hosts into three distinct clades, i.e. *T. leonina* isolates from wild felid hosts were in the same clade; *T. leonina* isolates from canid hosts including dogs and wolves formed another clade; while *T. leonina* isolates from foxes grouped together in a third clade. Our phylogenies (BI/ML) based on either ITS, *cox*2, *nad*1, *cox*2 plus *nad*1 or a combination of the nuclear (ITS) and mitochondrial (*cox*2 + *nad*1) data supported this split in which *T. leonina* specimens clustered into three subclades depending on their host species, i.e. foxes, wolfs and dogs and wild felids (including lions, tigers and lynxes; Figs. 2, 3). Such species complex phenomenon was also described in whipworms of primates. Thus, Liu et al. [62] and Hawash et al. [63] proposed that there may be several *Trichuris* species parasitizing primates, with some species only infecting non-human primates and others infecting both non-human primates and humans, on the basis of comparative mitogenomics. Variation in host range mirrors the interaction of trends in generalisation and specialization, the opportunity for host colonisation and the capacity to establish infection as reflected in outcomes for ecological fitting in sloppy fitness space [64, 65]. Moreover, faunal mixing and the potential for exchange can be also facilitated in zoopark environments that may bring phylogenetically disparate assemblages of hosts and their parasites into proximity [66]. For parasites with a direct life-cycle such as *Toxascaris*, this establishes the potential for considerable opportunities for host colonisation that may not be apparent in natural settings, and such extensive colonisation processes may further result in the possibility of new disease through the exchange of parasites over time. In addition, there were four species-specific non-synonymous base substitutions detected in *cox*2 and *nad*1 genes of *T. leonina* in the South China tiger, Eurasian lynx, African lion, Amur tiger and Bengal tiger (see Additional file 2: Figure S1, Additional file 3: Figure S2) that were further confirmed to be fixed after homologous comparisons with other congeneric species including that in the wolf.

As a part of epidemiological surveys of captive wild canids and felids in the southwestern zoos of China, this study focused on species diversity among *Toxocara*/*Toxascaris* spp. and raised some questions: (i) what routes are being possibly used for parasite introduction and establishment (natural or cross infections); (ii) what approaches are the best choices for surveillance of parasite infections, especially when various cryptic species may be involved; (iii) what strategies are the most reasonable interventions for prevention and control of parasite transport in the space-limited and artificial zoos; and (iv) what issues are the limits and facilitators for zoonotic risk. Given the fact that the recognised host range (defined by opportunity) of one parasite is almost always a subset of the actual host range (defined by capacity to use host-based resources), to some extent, this study also sets a quasi-experimental example to illustrate the roles of isolation and ecological fitting in expansions of host range. Such processes are most often related emerging infectious diseases in the context of ecological disruption, suggesting direct lessons about the potential *versus* realised host range for *Toxocara*/*Toxascaris* spp. and the risk of zoonotic infections. Therefore, it is urgent and necessary to clarify the species of *Toxocara*/*Toxascaris* which infect wild and domestic canids and felids in order to uncover transmission routes and develop suitable prevention and control measures.

## Conclusions

In the present study, based on the combined analysis of the nuclear and mitochondrial datasets, we suggest that at least three genetically distinct ascaridoid lineages are parasitizing wild canine and feline animals, i.e. *T. canis* in the arctic fox and red fox, *T. cati* in the jungle cat, leopard cat and Asian golden cat and *T. leonina* in the wolf, South China tiger, Eurasian lynx, African lion, Amur tiger and Bengal tiger. Further evidence derived from genetic distance analysis and phylogenies showed that there was a separation of *T. leonina* from different hosts, followed by the pattern that isolates from wild felids were in one subclade; isolates from dogs and wolves in another subclade and isolates from foxes in a third subclade, supporting a complex of *T. leonina* infecting these hosts. Of course, additional confirmation by further broad sampling and extensive morphological and genetic comparisons are required. Taken together, the results presented here yield new molecular insights into the classification, phylogenetic relationships and epidemiological importance of ascaridoids from wild canids and felids and also highlight the complex of the taxonomy and genetics of *Toxascaris* in their wild and domestic carnivorous hosts.

## Supplementary information

**Additional file 1: Table S1.** Nucleotide variability of the nuclear ITS and mitochondrial *cox*2 and *nad*1 genes of *Toxocara*/*Toxascaris* spp. identified in this study. *Abbreviations*: C, conserved sites; V, variable sites; Pi, parsimony-informative sites; S, singleton sites.

**Additional file 2: Figure S1.** A simultaneous alignment of nucleotide and amino acid sequences of partial mitochondrial *cox*1 genes of 11 representative isolates of *Toxocara*/*Toxascaris* identified in this study and other related ascaridoid species.

**Additional file 3: Figure S2.** A simultaneous alignment of nucleotide and amino acid sequences of partial mitochondrial *nad*1 genes of 11 representative isolates of *Toxocara*/*Toxascaris* identified in this study and other related ascaridoid species.

**Additional file 4: Table S2.** Estimates of evolutionary distance between ascaridoid species recovered from different host species using the nuclear ITS. Evolutionary distances between 11 wild animals included in this study are highlighted in bold for ITS-based estimates. Given almost identical nucleotide sequences of ITS regions in worms from the same host species, 11 representative specimens were used to calculate evolutionary distances using a maximum composite likelihood model.

**Additional file 5: Table S3.** Estimates of evolutionary distance between ascaridoid species recovered from different host species using the mitochondrial *cox*2 (below diagonal) and *nad*1 (above diagonal). Evolutionary distances between 11 wild animals included in this study are highlighted in bold for *cox*2- and *nad*1-based estimates, respectively. Given almost identical nucleotide sequences of *cox*2 or *nad*1 gene in worms from the same host species, 11 representative specimens were used to calculate evolutionary distances using a maximum composite likelihood model.

## Data Availability

Data supporting the conclusions of this article are included within the article and its additional files. Nucleotide sequences reported in this article are available in the GenBank database under the accession numbers JF837169-JF837179 and MK309890-MK309928 for ITS, JF780952-JF792252 and MK317996-MK318034 for *cox*2 and JF833955-JF833965 and MK318035-MK318073 for *nad*1.

## Abbreviations

ITS: internal transcribed spacer region
ITS2: second internal transcribed spacer region
*cox2*: cytochrome *c* oxidase subunit 2
*cox1*: cytochrome *c* oxidase subunit 1
*nad*1: NADH dehydrogenase subunit 1
*nad*4: NADH dehydrogenase subunit 4
rDNA: nuclear ribosomal DNA
mtDNA: mitochondrial DNA
gDNA: genomic DNA
VLM: visceral larva migrans
OLM: ocular larva migrans
EME: eosinophilic meningoencephalitis
CT: covert toxocariasis
NT: neurological toxocariasis
PCR: polymerase chain reaction
K2P: Kimura 2-parameter
BI: Bayesian inference
ML: maximum likelihood
GTR: general time reversible
indels: insertions/deletions

## References

[1] Okulewicz A, Perec-Matysiak A, Buńkowska K, Hildebrand J (2012). *Toxocara canis*, *Toxocara cati* and *Toxascaris leonina* in wild and domestic carnivores. Helminthologia.

[2] Ma G, Holland CV, Wang T, Hofmann A, Fan CK, Maizels RM, Hotez PJ, Gasser RB (2018). Human toxocariasis. Lancet Infect Dis.

[3] Moreira GM, Telmo Pde L, Mendonça M, Moreira AN, McBride AJ, Scaini CJ, Conceição FR (2014). Human toxocariasis: current advances in diagnostics, treatment, and interventions. Trends Parasitol.

[4] Holland C (2017). Knowledge gaps in the epidemiology of *Toxocara*: the enigma remains. Parasitology.

[5] Gasser RB (2013). A perfect time to harness advanced molecular technologies to explore the fundamental biology of *Toxocara* species. Vet Parasitol.

[6] Chen J, Zhou DH, Nisbet AJ, Xu MJ, Huang SY, Li MW (2012). Advances in molecular identification, taxonomy, genetic variation and diagnosis of *Toxocara* spp. Infect Genet Evol.

[7] Hajipour N (2019). A survey on the prevalence of *Toxocara cati*, *Toxocara canis* and *Toxascaris leonina* eggs in stray dogs and cats’ faeces in Northwest of Iran: a potential risk for human health. Trop Biomed.

[8] Macpherson CN (2013). The epidemiology and public health importance of toxocariasis: a zoonosis of global importance. Int J Parasitol.

[9] Lee AC, Schantz PM, Kazacos KR, Montgomery SP, Bowman DD (2010). Epidemiologic and zoonotic aspects of ascarid infections in dogs and cats. Trends Parasitol.

[10] Le TH, Anh NT, Nguyen KT, Nguyen NT, Thuy do TT, Gasser RB (2016). *Toxocara malaysiensis* infection in domestic cats in Vietnam—an emerging zoonotic issue?. Infect Genet Evol.

[11] Jin YC, Li XY, Liu JH, Zhu XQ, Liu GH (2019). Comparative analysis of mitochondrial DNA datasets indicates that *Toxascaris leonina* represents a species complex. Parasit Vectors.

[12] Fogt-Wyrwas R, Dabert M, Jarosz W, Rząd I, Pilarczyk B, Mizgajska-Wiktor H (2019). Molecular data reveal cryptic speciation and host specificity in *Toxascaris leonina* (Nematoda: Ascarididae). Vet Parasitol.

[13] Gasser R, Zhu XQ, Hu M, Jacobs DE, Chilton NB, Holland CV, Smith HV (2006). Molecular genetic characterization of members of the genus *Toxocara*—taxonomic, population genetic and epidemiological considerations. *Toxocara*: the enigmatic parasite.

[14] Fogt-Wyrwas R, Jarosz W, Rzad I, Pilarczyk B, Mizgajska-Wiktor H (2016). Variability in sequences of mitochondrial *cox*1 and *nadh*1 genes in *Toxocara canis*, *Toxocara cati*, and *Toxascaris leonina* (Nematoda: Toxocaridae) from different hosts. Ann Parasitol.

[15] Li M, Lin R, Chen H, Sani R, Song H, Zhu X (2007). PCR tools for the verification of the specific identity of ascaridoid nematodes from dogs and cats. Mol Cell Probes.

[16] Li MW, Lin RQ, Song HQ, Sani RA, Wu XY, Zhu XQ (2008). Electrophoretic analysis of sequence variability in three mitochondrial DNA regions for ascaridoid parasites of human and animal health significance. Electrophoresis.

[17] Zhu X, Jacobs D, Chilton N, Sani R, Cheng N, Gasser R (1998). Molecular characterization of a *Toxocara* variant from cats in Kuala Lumpur, Malaysia. Parasitology.

[18] Jacobs DE, Zhu X, Gasser RB, Chilton NB (1997). PCR-based methods for identification of potentially zoonotic ascaridoid parasites of the dog, fox and cat. Acta Trop.

[19] Vega R, Prous CG, Krivokapich S, Gatti G, Brugni N, Semenas L (2018). Toxocariasis in Carnivora from Argentinean Patagonia: species molecular identification, hosts, and geographical distribution. Int J Parasitol Parasites Wildl.

[20] Hu M, Gasser RB (2006). Mitochondrial genomes of parasitic nematodes—progress and perspectives. Trends Parasitol.

[21] Mikaeili F, Mirhendi H, Mohebali M, Hosseini M, Sharbatkhori M, Zarei Z (2015). Sequence variation in mitochondrial *cox*1 and *nad*1 genes of ascaridoid nematodes in cats and dogs from Iran. J Helminthol.

[22] Nadler SA, Hudspeth DS (2000). Phylogeny of the Ascaridoidea (Nematoda: Ascaridida) based on three genes and morphology: hypotheses of structural and sequence evolution. J Parasitol.

[23] Li MW, Zhu XQ, Gasser RB, Lin RQ, Sani RA, Lun ZR (2006). The occurrence of *Toxocara malaysiensis* in cats in China, confirmed by sequence-based analyses of ribosomal DNA. Parasitol Res.

[24] Li Y, Niu L, Wang Q, Zhang Z, Chen Z, Gu X (2012). Molecular characterization and phylogenetic analysis of ascarid nematodes from twenty-one species of captive wild mammals based on mitochondrial and nuclear sequences. Parasitology.

[25] Sheng ZH, Chang QC, Tian SQ, Lou Y, Zheng X, Zhao Q (2012). Characterization of *Toxascaris leonina* and *Tococara canis* from cougar (*Panthera leo*) and common wolf (*Canis lupus*) by nuclear ribosomal DNA sequences of internal transcribed spacers. Afr J Microbiol Res.

[26] Jenkins EJ (2020). *Toxocara* spp. in dogs and cats in Canada. Adv Parasitol.

[27] Taylor MA, Coop RL, Wall RL (2007). Veterinary parasitology.

[28] Warren G (1970). Studies on the morphology and taxonomy of the genera *Toxocara* Stiles, 1905 and *Neoascaris* Travassos, 1927. Zool Anz.

[29] Thompson JD, Gibson TJ, Plewniak F, Jeanmougin F, Higgins DG (1997). The CLUSTAL_X windows interface: flexible strategies for multiple sequence alignment aided by quality analysis tools. Nucleic Acids Res.

[30] Tamura K, Stecher G, Peterson D, Filipski A, Kumar S (2013). MEGA6: molecular evolutionary genetics analysis version 6.0. Mol Biol Evol.

[31] Ronquist F, Teslenko M, van der Mark P, Ayres DL, Darling A, Höhna S (2012). MrBayes 3.2: efficient Bayesian phylogenetic inference and model choice across a large model space. Syst Biol.

[32] Guindon S, Dufayard JF, Lefort V, Anisimova M, Hordijk W, Gascuel O (2010). New algorithms and methods to estimate maximum-likelihood phylogenies: assessing the performance of PhyML 3.0. Syst Biol.

[33] Darriba D, Taboada GL, Doallo R, Posada D (2012). jModelTest 2: more models, new heuristics and parallel computing. Nat Methods.

[34] Li K, Yang F, Abdullahi A, Song M, Shi X, Wang M (2016). Sequence analysis of mitochondrial genome of *Toxascaris leonina* from a South China Tiger. Korean J Parasitol.

[35] Liu GH, Zhou DH, Zhao L, Xiong RC, Liang JY, Zhu XQ (2014). The complete mitochondrial genome of *Toxascaris leonina*: comparison with other closely related species and phylogenetic implications. Infect Genet Evol.

[36] Xie Y, Niu L, Zhao B, Wang Q, Nong X, Chen L (2013). Complete mitochondrial genomes of chimpanzee-and gibbon-derived *Ascaris* isolated from a zoological garden in southwest China. PLoS ONE.

[37] Xie Y, Zhang Z, Wang C, Lan J, Li Y, Chen Z (2011). Complete mitochondrial genomes of *Baylisascaris schroederi*, *Baylisascaris ailuri* and *Baylisascaris transfuga* from giant panda, red panda and polar bear. Gene.

[38] Bradley CA, Altizer S (2007). Urbanization and the ecology of wildlife diseases. Trends Ecol Evol.

[39] Fryxell JM, Sinclair AR, Caughley G (2014). Wildlife ecology, conservation, and management.

[40] Mackenstedt U, Jenkins D, Romig T (2015). The role of wildlife in the transmission of parasitic zoonoses in peri-urban and urban areas. Int J Parasitol Parasites Wildl.

[41] Xie Y, Hoberg EP, Yang Z, Urban JF, Yang G (2017). *Ancylostoma ailuropodae* n. sp. (Nematoda: Ancylostomatidae), a new hookworm parasite isolated from wild giant pandas in Southwest China. Parasites Vectors.

[42] Antolová D, Reiterová K, Miterpáková M, Stanko M, Dubinský P (2004). Circulation of *Toxocara* spp. in suburban and rural ecosystems in the Slovak Republic. Vet Parasitol.

[43] Criado-Fornelio A, Gutierrez-Garcia L, Rodriguez-Caabeiro F, Reus-Garcia E, Roldan-Soriano M, Diaz-Sanchez M (2000). A parasitological survey of wild red foxes (*Vulpes vulpes*) from the province of Guadalajara, Spain. Vet Parasitol.

[44] Popiołek M, Szczęsna J, Nowak S, Mysłajek RW (2007). Helminth infections in faecal samples of wolves *Canis lupus* L. from the western Beskidy Mountains in southern Poland. J Helminthol.

[45] Smith G, Gangadharan B, Taylor Z, Laurenson M, Bradshaw H, Hide G (2003). Prevalence of zoonotic important parasites in the red fox (*Vulpes vulpes*) in Great Britain. Vet Parasitol.

[46] Torres J, Garcia-Perea R, Gisbert J, Feliu C (1998). Helminth fauna of the Iberian lynx, *Lynx pardinus*. J Helminthol.

[47] Blouin MS, Yowell CA, Courtney CH, Dame JB (1998). Substitution bias, rapid saturation, and the use of mtDNA for nematode systematics. Mol Biol Evol.

[48] Hu M, Chilton NB, Gasser RB (2004). The mitochondrial genomics of parasitic nematodes of socio-economic importance: recent progress, and implications for population genetics and systematics. Adv Parasitol.

[49] Magi M, Macchioni F, Dell’Omodarme M, Prati M, Calderini P, Gabrielli S (2009). Endoparasites of red fox (*Vulpes vulpes*) in central Italy. J Wildl Dis.

[50] Mørk T, Ims RA, Killengreen ST (2019). Rodent population cycle as a determinant of gastrointestinal nematode abundance in a low-arctic population of the red fox. Int J Parasitol Parasites Wildl.

[51] Saeed I, Maddox-Hyttel C, Monrad J, Kapel CM (2006). Helminths of red foxes (*Vulpes vulpes*) in Denmark. Vet Parasitol.

[52] Stien A, Voutilainen L, Haukisalmi V, Fuglei E, Mørk T, Yoccoz N (2010). Intestinal parasites of the Arctic fox in relation to the abundance and distribution of intermediate hosts. Parasitology.

[53] Reperant LA, Hegglin D, Fischer C, Kohler L, Weber J-M, Deplazes P (2007). Influence of urbanization on the epidemiology of intestinal helminths of the red fox (*Vulpes vulpes*) in Geneva, Switzerland. Parasitol Res.

[54] Strube C, Heuer L, Janecek E (2013). *Toxocara* spp. infections in paratenic hosts. Vet Parasitol.

[55] Borecka A, Gawor J, Zieba F (2013). A survey of intestinal helminths in wild carnivores from the Tatra National Park, southern Poland. Ann Parasitol.

[56] Meijer T, Mattsson R, Angerbjörn A, Osterman-Lind E, Fernández-Aguilar X, Gavier-Widén D (2011). Endoparasites in the endangered Fennoscandian population of arctic foxes (*Vulpes lagopus*). Eur J Wildl Res.

[57] Millán J, Casanova JC (2009). High prevalence of helminth parasites in feral cats in Majorca Island (Spain). Parasitol Res.

[58] Zibaei M, Sadjjadi SM, Sarkari B (2007). Prevalence of *Toxocara cati* and other intestinal helminths in stray cats in Shiraz, Iran. Trop Biomed.

[59] Radwan NA, Khalil AI, El Mahi RA (2009). Morphology and occurrence of species of *Toxocara* in wild mammal populations from Egypt. Comp Parasitol.

[60] Napoli E, Anile S, Arrabito C, Scornavacca D, Mazzamuto MV, Gaglio G (2016). Survey on parasitic infections in wildcat (*Felis silvestris silvestris* Schreber, 1777) by scat collection. Parasitol Res.

[61] Xie Y, Li H, Wang C, Li Y, Liu Y, Meng X (2019). Characterization of the complete mitochondrial genome sequence of the dog roundworm *Toxascaris leonina* (Nematoda, Ascarididae) from China. Mitochondrial DNA B Resour.

[62] Liu GH, Gasser RB, Nejsum P, Wang Y, Chen Q, Song HQ (2013). Mitochondrial and nuclear ribosomal DNA evidence supports the existence of a new *Trichuris* species in the endangered françois’ leaf-monkey. PLoS ONE.

[63] Hawash MB, Andersen LO, Gasser RB, Stensvold CR, Nejsum P (2015). Mitochondrial genome analyses suggest multiple *Trichuris* species in humans, baboons, and pigs from different geographical regions. PLoS Negl Trop Dis.

[64] Agosta SJ, Janz N, Brooks DR (2010). How specialists can be generalists: resolving the “parasite paradox” and implications for emerging infectious disease. Zoologia.

[65] Araujo SB, Braga MP, Brooks DR, Agosta SJ, Hoberg EP, von Hartenthal FW (2015). Understanding host-switching by ecological fitting. PLoS ONE.

[66] Xie Y, Zhao B, Hoberg EP, Li M, Zhou X, Gu X (2018). Genetic characterisation and phylogenetic status of whipworms (*Trichuris* spp.) from captive non-human primates in China, determined by nuclear and mitochondrial sequencing. Parasites Vectors.

[67] Hoberg EP, Burek-Huntington K, Beckmen K, Camp LE, Nadler SA (2018). Transuterine infection by *Baylisascaris transfuga*: neurological migration and fatal debilitation in sibling moose calves (*Alces alces gigas*) from Alaska. Int J Parasitol Parasites Wildl.

[68] Arizono N, Yoshimura Y, Tohzaka N, Yamada M, Tegoshi T, Onishi K (2010). Ascariasis in Japan: is pig-derived *Ascaris* infecting humans. Jpn J Infect Dis.

[69] Li MW, Lin RQ, Song HQ, Wu XY, Zhu XQ (2008). The complete mitochondrial genomes for three *Toxocara* species of human and animal health significance. BMC Genomics.

[70] He X, Lv MN, Liu GH, Lin RQ (2018). Genetic analysis of *Toxocara cati* (Nematoda: Ascarididae) from Guangdong province, subtropical China. Mitochondrial DNA A DNA Mapp Seq Anal.

[71] Jex AR, Waeschenbach A, Littlewood DTJ, Hu M, Gasser RB (2008). The mitochondrial genome of *Toxocara canis*. PLoS Negl Trop Dis.

[72] Wickramasinghe S, Yatawara L, Agatsuma T (2014). *Toxocara canis* (Ascaridida: Nematoda): mitochondrial gene content, arrangement and composition compared with other *Toxocara* species. Sri Lankan J Infect Dis.

[73] Choobineh M, Mikaeili F, Sadjjadi S, Ebrahimi S, Iranmanesh S (2019). Molecular characterization of *Toxocara* spp. eggs isolated from public parks and playgrounds in Shiraz, Iran. J Helminthol.

[74] Li K, Lan Y, Luo H, Zhang H, Liu D, Zhang L (2016). Prevalence, associated risk factors, and phylogenetic analysis of *Toxocara vitulorum* infection in yaks on the Qinghai Tibetan plateau, China. Korean J Parasitol.

[75] Wickramasinghe S, Yatawara L, Rajapakse R, Agatsuma T (2009). *Toxocara vitulorum* (Ascaridida: Nematoda): mitochondrial gene content, arrangement and composition compared with other *Toxocara* species. Mol Biochem Parasitol.

[76] Choi Y, Mason S, Ahlborn M, Zscheile B, Wilson E (2017). Partial molecular characterization of the mitochondrial genome of *Baylisascaris columnaris* and prevalence of infection in a wild population of striped skunks. Int J Parasitol Parasites Wildl.

[77] Lin Q, Li H, Gao M, Wang X, Ren W, Cong M (2012). Characterization of *Baylisascaris schroederi* from Qinling subspecies of giant panda in China by the first internal transcribed spacer (ITS-1) of nuclear ribosomal DNA. Parasitol Res.

[78] Li K, Luo H, Zhang H, Mehmood K, Shahzad M, Zhang L (2018). Analysis of the internal transcribed spacer region of *Ascaris suum* and *Ascaris lumbricoides* derived from free range Tibetan pigs. Mitochondrial DNA A DNA Mapp Seq Anal.

[79] Wolstenholme DR, Okimoto R, Macfarlane JL (1994). Nucleotide correlations that suggest tertiary interactions in the TV-replacement loop-containing mitochondrial tRNAs of the nematodes, *Caenorhabditis elegans* and *Ascaris suum*. Nucleic Acids Res.

[80] Park YC, Kim W, Park JK (2011). The complete mitochondrial genome of human parasitic roundworm, *Ascaris lumbricoides*. Mitochondrial DNA.

