# Supplementary material for: Molecular characterization of ascaridoid parasites from captive wild carnivores in China using ribosomal and mitochondrial sequences
Source: Parasit Vectors. 2020 Jul 29;13:382. doi: 10.1186/s13071-020-04254-4 (PMC7391581; doi:10.1186/s13071-020-04254-4)
Supplement: Supplementary file 2 — Additional file 2: Figure S1. A simultaneous alignment of nucleotide and amino acid sequences of partial mitochondrial cox1 genes of 11 representative isolates of Toxocara/Toxascaris identified in this study and other related ascaridoid species. [file 13071_2020_4254_MOESM2_ESM.docx]

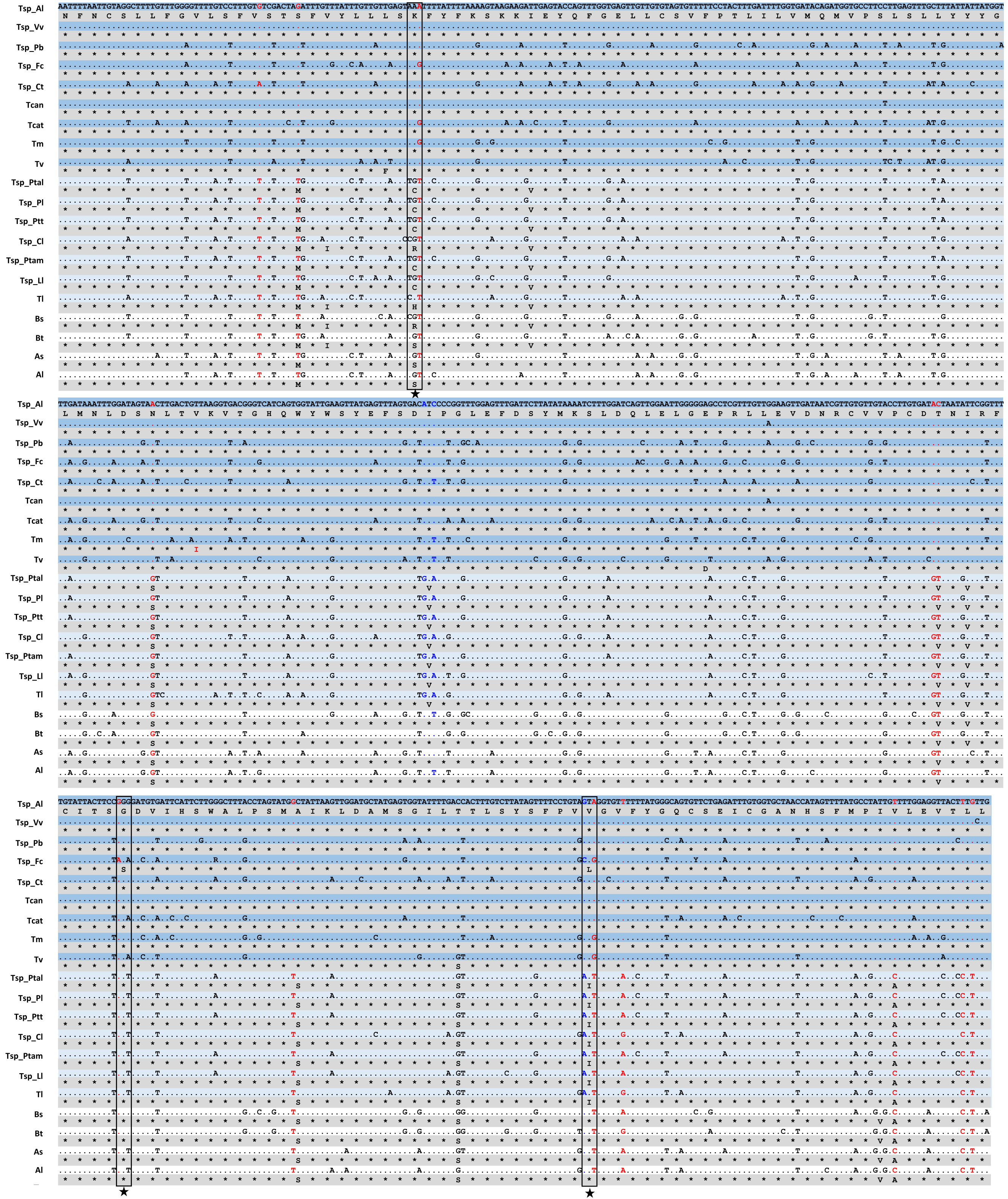


**Additional file 2: Figure S1.** A simultaneous alignment of nucleotide and amino acid sequences of partial mitochondrial *cox*1 genes of 11 representative isolates of *Toxocara*/*Toxascaris* identified in this study and other related ascaridoid species. Building on the alignment of nucleotide sequences, the corresponding amino acid sequences inferred according to the Invertebrate Mitochondrial Code were added and aligned. Regions of identity in either nucleotide (.) or amino-acid (*) are indicated. The variable base loci unique for *Toxocara* spp. are highlighted in red and for *Toxascaris* spp. highlighted in blue. Further, these variable base loci unique for *Toxocara*/*Toxascaris* are subjected to detection of the non-synonymous substitutions, and a total of 3 amino-acid changes: K/R/S (Va/Arg/Ser)→C(Ilu), G(Gly)→S(Ser) and V/I(Val/Ilu)→L(Leu) are observed and targeted with a black star. Species abbreviations and GenBank accession numbers (in parentheses) are indicated as follows: Tsp_Al, *Toxocara*/*Toxascaris* sp. from *A. lagopus* (MK318032); Tsp_Vv, *Toxocara*/*Toxascaris* sp. from *V. vulpes* (MK318034); Tsp_Pb, *Toxocara*/*Toxascaris* sp. from *P. bengalensis* (JF792244); Tsp_Fc, *Toxocara*/*Toxascaris* sp. from *F. chaus* (MK318031); Tsp_Ct, *Toxocara*/*Toxascaris* sp. from *C. temmincki* (MK318007); Tsp_Ptal, *Toxocara*/*Toxascaris* sp. from *P. tigris altaica* (MK317996); Tsp_Pl, *Toxocara*/*Toxascaris* sp. from *P. leo* (MK318011); Tsp_Ptt, *Toxocara*/*Toxascaris* sp. from *P. tigris tigris* (MK318001); Tsp_Cl, *Toxocara*/*Toxascaris* sp. from *Canis lupus* (JF792247); Tsp_Ptam, *Toxocara*/*Toxascaris* sp. from *P. tigris amoyensis* (MK318025); Tsp_Ll, *Toxocara*/*Toxascaris* sp. from *L. lynx* (MK318009); *T. canis* (JN593098); *T. cati* (NC_010773); *T. malaysiensis* (AM412316); *T. vitulorum* (FJ664617); *T. leonina* (NC_023504); *Baylisascaris schroederi* (HQ671081); *B. transfuga* (NC_015924); *Ascaris suum* (NC_001327); and *A. lumbricoides* (JN801161).
